# Deciphering protein prenylation in endocytic trafficking in *Toxoplasma gondii*

**DOI:** 10.1128/mbio.00283-24

**Published:** 2024-02-26

**Authors:** Vern B. Carruthers, Zhicheng Dou

**Affiliations:** 1 Department of Microbiology and Immunology, University of Michigan Medical School, Ann Arbor, Michigan, USA; 2 Department of Biological Sciences, Clemson University, Clemson, South Carolina, USA; Albert Einstein College of Medicine, Bronx, New York, USA

**Keywords:** *Toxoplasma gondii*, protozoan, endocytosis, host-pathogen interaction

## Abstract

*Toxoplasma gondii* is a widespread intracellular protozoan pathogen infecting virtually all warm-blooded animals. This parasite acquires host-derived resources to support its replication inside a membrane-bound parasitophorous vacuole within infected host cells. Previous research has discovered that *Toxoplasma* actively endocytoses host proteins and transports them to a lysosome-equivalent structure for digestion. However, few molecular determinants required for trafficking of host-derived material within the parasite were known. A recent study (Q.-Q. Wang, M. Sun, T. Tang, D.-H. Lai, et al., mBio 14:e01309-23, 2023, https://doi.org/10.1128/mbio.01309-23) identified a critical role for membrane anchoring of proteins via prenylation in the trafficking of endocytosed host proteins by *Toxoplasma*, including an essential *Toxoplasma* ortholog of Rab1B. The authors also found that TgRab1 is crucial for protein trafficking of the rhoptry secretory organelles, indicating a dual role in endocytic and exocytic protein trafficking. This study sets the stage for further dissecting endomembrane trafficking in *Toxoplasma*, along with potentially exploiting protein prenylation as a target for therapeutic development.

## COMMENTARY

*Toxoplasma gondii* is known for its versatile ability to infect a diverse range of hosts, leading to the development of toxoplasmosis in both humans and animals (1). *Toxoplasma* hijacks the host cell’s plasma membrane to create a specialized intracellular replicative compartment termed the parasitophorous vacuole (PV) (2). This membrane-bound structure affords protection from host intrinsic defenses, but it is also a barrier across which the parasite must acquire resources from the infected cell. Previous work has shown that *Toxoplasma* uses three mechanisms to secure resources across the PV membrane (3, 4). First, it secretes proteins that form pores in the PV membrane to access small (<1,300 Da) nutrients (5, 6), which are subsequently imported into the parasite by membrane transporters. Second, it captures within the PV lipid-laden vesicles derived from several host organelles (7–9), mining them to acquire lipids, including cholesterol.

A third mechanism whereby *Toxoplasma* acquires resources is termed the ingestion pathway (10). In this pathway, the parasite exploits the host endosomal sorting complexes responsible for trafficking machinery to bud small vesicles containing host-derived proteins into the PV lumen (11). Once these vesicles reach the parasite plasma membrane, they have been suggested to enter a specialized endocytic structure termed the micropore as a site of endocytosis. Two recent studies independently identified the first molecular components of micropores (12, 13). After endocytosis, the ingested host proteins appear to navigate through the parasite’s endolysosomal system, including possibly transiting the trans-Golgi network (TGN) before entering the endosome-like compartment (ELC) (14), a mixture of vesicles displaying markers of both early and late endosomes. Finally, the host-derived proteins are delivered to a lysosome-equivalent organelle termed the plant-like vacuolar compartment (PLVAC), where they are degraded by cathepsins (10, 15), presumably to metabolize or recycle liberated amino acids. Notably, the *Toxoplasma* ingestion pathway broadly resembles *Plasmodium* spp. (malaria parasites) internalization of hemoglobin from infected erythrocytes for delivery to the parasite’s digestive vacuole.

In model systems, trafficking of endocytic material through the endolysosomal system critically relies on small Rab GTPases, including Rab5 for early endosomes and Rab7 for late endosomes (16). These Rab GTPases require prenylation, a covalent posttranslational modification involving the addition of a farnesyl or geranylgeranyl unsaturated lipid moiety to the C-terminal cysteine of a target protein, thereby anchoring it to a cellular membrane (17, 18). However, it remained a mystery how prenylation is involved in the trafficking of endocytosed proteins within the intracellular endolysosomal system of *Toxoplasma* parasites. A recent study published in *mBio* by Wang et al. (19) employed an alkyne-labeled click chemistry approach to identify the prenylome of *Toxoplasma* parasites. They subsequently used a host green fluorescent protein (GFP) acquisition assay to identify prenylated proteins involved in the endocytic trafficking of host proteins, including Rab1B, YKT6.1, Ras, and Rab8/10-like proteins. Using CRISPR-based gene knockout and knockdown strategies, together with high-resolution imaging techniques, the authors systemically defined the subcellular localizations and functions of prenylated proteins. The paper thereafter focused on *Toxoplasma* Rab1B due to its essential role. In other organisms, Rab1 orthologs control trafficking through the early secretory pathway, including endoplasmic reticulum (ER)-to-Golgi and intra-Golgi traffic (20). Rab1 also regulates macroautophagy by recruiting Atg1 to the pre-autophagosomal structure and tethering Atg9 vesicles for bulk autophagy (21). In *Toxoplasma*, TgRab1B was observed to localize in both TGN and ELC, suggesting it resides further along in the secretory system than in other organisms. Depletion of TgRab1B not only impaired delivery of host-derived protein to the PLVAC but also affected the delivery of proteins to the rhoptry secretory organelles and the plasma membrane, among other sites. These findings indicate TgRab1B plays a dual role in trafficking: conventionally for exocytic trafficking but unconventionally for endocytic trafficking. This dual role is consistent with the hybrid nature of the *Toxoplasma* endo/exocytic system, wherein endocytic and exocytic materials traffic through a shared organelle, the ELC (14). In addition, the chemical and genetic inhibition of prenylation reduced the abundance of TgRab1B, suggesting that prenylation is crucial for the stability of TgRab1B.

In summary, the findings from the study by Wang et al. deepen our understanding of the molecular mechanisms governing the trafficking of endocytosed host proteins in *Toxoplasma* by identifying a few prenylated proteins involved in the formation of transporting vesicles. Further research is needed to determine how the depletion of TgRab1B affects the integrity of the ELC to disrupt endo and exocytic traffic. It will also be interesting to determine if TgRab1B functions in *Toxoplasma* autophagy, which is crucial for long-term survival of the parasite during its chronic stage (22, 23). Overall, the identification of prenylated components suggests that lipidation of molecular switches may be a potential druggable pathway for treating toxoplasmosis.
